# Multi-omic, Single-Cell, and Biochemical Profiles of Astronauts Guide Pharmacological Strategies for Returning to Gravity

**DOI:** 10.1016/j.celrep.2020.108429

**Published:** 2020-11-25

**Authors:** Monica L. Gertz, Christopher R. Chin, Delia Tomoiaga, Matthew MacKay, Christina Chang, Daniel Butler, Ebrahim Afshinnekoo, Daniela Bezdan, Michael A. Schmidt, Christopher Mozsary, Ari Melnick, Francine Garrett-Bakelman, Brian Crucian, Stuart M.C. Lee, Sara R. Zwart, Scott M. Smith, Cem Meydan, Christopher E. Mason

**Affiliations:** 1 Department of Physiology and Biophysics, Weill Cornell Medicine, New York, NY 10021, USA; 2 Interdisciplinary Program in Neuroscience, George Mason University, Fairfax, VA 22030, USA; 3 The WorldQuant Initiative for Quantitative Prediction, Weill Cornell Medicine, New York, NY 10065, USA; 4 The HRH Prince Alwaleed Bin Talal Bin Abdulaziz Alsaud Institute for Computational Biomedicine, Weill Cornell Medicine, New York, NY 10021, USA; 5 Institute of Medical Virology and Epidemiology of Viral Diseases, University Hospital, Tübingen 72076, Germany; 6 Advanced Pattern Analysis and Countermeasures Group, Boulder, CO 80302, USA; 7 Sovaris Aerospace, Boulder, CO 80302, USA; 8 Department of Medicine, Weill Cornell Medicine, New York, NY 10021, USA; 9 Department of Medicine, University of Virginia, Charlottesville, VA 22908, USA; 10 Department of Biochemistry and Molecular Genetics, University of Virginia, Charlottesville, VA 22908, USA; 11 University of Virginia Cancer Center, Charlottesville, VA 22908, USA; 12 Human Health and Performance Directorate, NASA Johnson Space Center, Houston, TX 77058, USA; 13 KBR, Houston, TX 77058, USA; 14 Department of Preventive Medicine and Population Health, University of Texas Medical Branch, Galveston, TX 77555, USA; 15 The Feil Family Brain and Mind Research Institute, Weill Cornell Medicine, New York, NY 10065, USA; 16 Becton Dickinson & Co., Washington, DC 20001

## Abstract

The National Aeronautics and Space Administration (NASA) Twins Study created an integrative molecular profile of an astronaut during NASA’s first 1-year mission on the International Space Station (ISS) and included comparisons to an identical Earth-bound twin. The unique biochemical profiles observed when landing on Earth after such a long mission (e.g., spikes in interleukin-1 [IL-1]/6/10, c-reactive protein [CRP], C-C motif chemokine ligand 2 [CCL2], IL-1 receptor antagonist [IL-1ra], and tumor necrosis factor alpha [TNF-α]) opened new questions about the human body’s response to gravity and how to plan for future astronauts, particularly around initiation or resolution of inflammation. Here, single-cell, multi-omic (100-plex epitope profile and gene expression) profiling of peripheral blood mononuclear cells (PBMCs) showed changes to blood cell composition and gene expression post-flight, specifically for monocytes and dendritic cell precursors. These were consistent with flight-induced cytokine and immune system stress, followed by skeletal muscle regeneration in response to gravity. Finally, we examined these profiles relative to 6-month missions in 28 other astronauts and detail potential pharmacological interventions for returning to gravity in future missions.

## In Brief

Gertz et al. present a re-analysis of the landing data from the NASA Twins Study, suggesting that the biochemical signature reflects muscle regeneration after atrophy rather than a detrimental inflammatory response. This is mediated through muscle-derived IL-6 anti-inflammatory cascades. Single-cell analysis supports this role. Potential pharmacological interventions are also discussed.

## Graphical Abstract

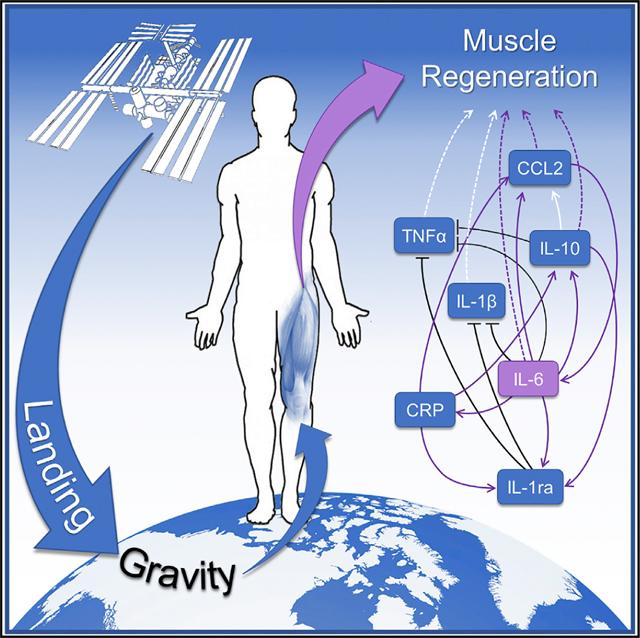

## INTRODUCTION

Landing on a planet after transit in space is a complex event for the human body, leading to a broad range of physiological and psychological stressors. The National Aeronautics and Space Administration (NASA) Twins Study (Garrett-Bakelman et al., 2019) resulted in the first-ever multi-omic profile of an astronaut after almost a year (340 days) on the International Space Station (ISS), while leveraging a comparison to his monozygotic Earth-bound twin. More than 300 samples were collected across 19 time points before, during, and after spaceflight, and data were generated across myriad modalities of human and microbial biology, including isolations of stool, saliva, skin, urine, plasma, peripheral blood mononuclear cells (PBMCs), and immune cells that were CD4^+^, CD8^+^, and CD19^+^ enriched and lymphocyte depleted (LD). The study used a wide battery of methods: short-read and long-read whole-genome sequencing (WGS), whole-genome bisulfite sequencing (WGBS), RNA sequencing (poly(A), ribo-, microRNA [miRNA]), shotgun metagenome sequencing, 16S rRNA sequencing, targeted proteomics (liquid chromatography-mass spectrometry [LC-MS]), untargeted proteomics (PECAN, MaxQuant), targeted metabolomics (gas chromatography [GC]-MS), mitochondrial respiration (SeaHorse), oxidative state (electron paramagnetic resonance [EPR]), T cell and B cell receptor (TCR/BCR) profiling, cognition tests, vascular measures, and health biometrics (nutrition, height, weight) (Garrett-Bakelman et al., 2019). Previous results identified a significant increase upon landing of five markers: interleukin-6 (IL-6), c-reactive protein (CRP), IL-10, C-C motif chemokine ligand 2 (CCL2/monocyte chemoattractant protein-1 [MCP1]), and IL-1 receptor antagonist (IL-1ra), which is followed immediately by a decrease in markers that were elevated during flight. These included tumor necrosis factor α (TNF-α), IL-1α, and IL-1β, which are normally associated with immune dysregulation but are also involved in bone metabolism and early stages of muscle regeneration.

To narrow down the roles that these inflammatory proteins and epitopes play during landing, we considered gravity as one of the most prominent changes to affect flight subject (TW) on landing, although we cannot disregard other factors (e.g., environment). Based on previous studies performed on astronauts following 6-month missions, it was determined that in-flight countermeasures were insufficient to fully replicate or replace the daily mechanical loading exerted by gravity (Genc et al., 2010), which resulted in decreased muscle volume and strength, particularly in the lower extremities (Akima et al., 2000; Trappe et al., 2009; Gopalakrishnan et al., 2010; Smith et al., 2012a; Korth, 2015; Smith, 2018; Lee et al., 2020b). Moreover, muscle and bone deterioration are time-dependent processes (Winnard et al., 2019; Ko et al., 2020). Therefore, reintroduction to Earth’s gravity after a year of weightlessness would likely cause greater reloading injury and inflammation during muscle regeneration and growth than might be seen after 6 months in space (Fitts et al., 2000; Matsumoto et al., 2011; Stein, 2013; Smith, 2018). The thorough biochemical profiling performed in the Twins Study has captured this post-landing phenomenon (Garrett-Bakelman et al., 2019).

Here, we generated single-cell, multi-omic (protein and RNA) data from the twin astronauts (TW and ground subject [HR]) and examined in greater detail the unique biochemical profiles expressed upon return to unit gravity (1g). Single-cell epitope and expression analysis of peripheral blood monocytes (PBMCs) showed elevated levels of classical monocytes (CD14^+^, CD16^−^, pro-inflammatory) on landing compared with pre-flight values, which proceeded to decrease post-flight as non-classical monocytes (CD14^−^, CD16^+^, anti-inflammatory) increased. Many of these proteins are pleiotropic, exerting many functions throughout the body, and they are often associated with initiation or resolution of detrimental inflammatory responses. In light of the self-reported flu-like symptoms, skin inflammation, and discomfort experienced by TW on landing, the question arises as to whether such symptoms should be treated pharmacologically to attenuate them for future missions; and if so, how? In this analysis, we describe the major effects and interactions of these proteins with respect to the process of muscle regeneration as well as examine possible pharmacological interventions for future astronauts.

## RESULTS

### Biochemical Response to Landing on Earth

**Figure 1.**
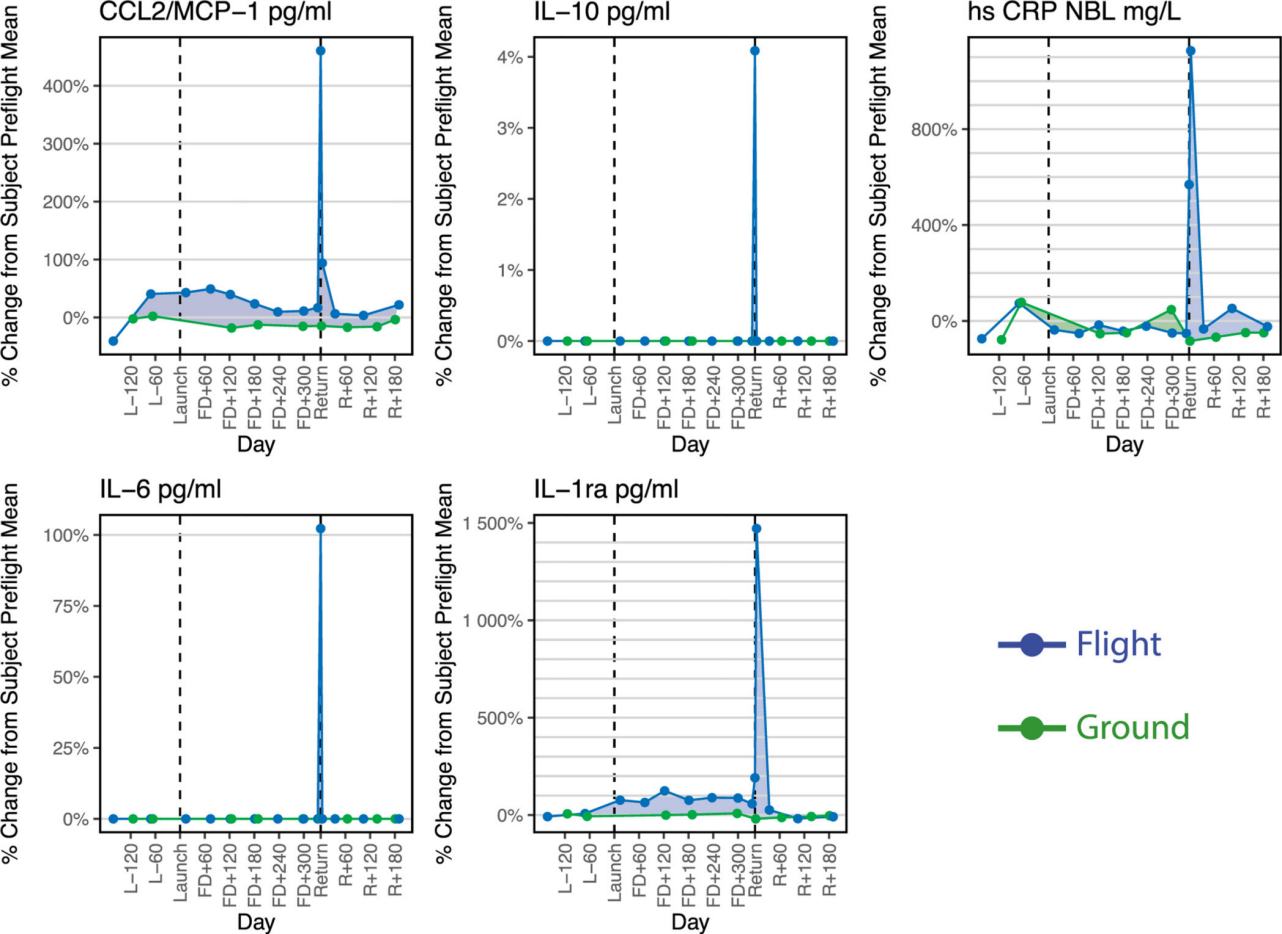
Biochemical Response to the Return to Gravity The biochemical profiles expressed upon return to Earth, showing five established inflammation markers with a significant increase upon landing: C-C motif chemokine ligand 2 (CCL2)/MCP1, interleukin-10 (IL-10), c-reactive protein (CRP), IL-6, and IL-1 receptor antagonist (IL-1ra). The y axis is the percent difference from the mean of each subject’s pre-flight value plotted over time, whereas the x axis is number of days during the mission. Blue lines are for the flight subject (TW), and green lines are for the ground subject (HR). (Garrett-Bakelman et al., 2019) See also Figure S1.

Biochemical profiling of the flight subject TW revealed a clear molecular signature in response to his return to 1g (Figure 1), most notably for the samples collected on day zero (R0) at Johnson Space Center (JSC) crew quarters, ~24 h after de-orbit. During this time, TW engaged in some ambulatory activities, but slept for the greater part of the journey from Kazakhstan to JSC. A second set of samples was then collected on R+5 (Garrett-Bakelman et al., 2019; Lee et al., 2020a). The cytokines depicted in Figure 1 showed relatively stable levels in TW, with a significant spike immediately after landing, with increases at R0 and R+5 in IL-6 (100%), CRP (1,150%), IL-10 (4%), CCL2/MCP1 (475%), and IL-1ra (1,500%), compared to pre-flight means (ANOVA, *q* < 0.001) (Garrett-Bakelman et al., 2019). These cytokines are normally associated with inflammatory immune responses, and by R+60, all biochemical markers had returned to pre-flight levels. These cytokines are also associated with muscle regeneration and indicate a physiological response to muscle exertion in the presence of gravity as opposed to a purely detrimental inflammatory response.

Interestingly, these changes are consistent with changes in other astronauts after shorter (up to 6-month-duration) missions (Figure S1) (Crucian et al., 2014). IL-6, IL-10, CCL2, and IL-1ra all showed major increases upon landing compared with pre-flight median levels for each astronaut, although TW showed a more pronounced elevation of these markers after his 12-month mission than the set of 28 astronauts returning from 6-month missions. It should be noted that the IL-6 and IL-10 results for TW were below the assay detection limits for pre-flight and post-flight samples but close to the upper limit of detection for the return time point in the comparable assay plotted in Figure S1, making absolute quantification difficult.

### Pathway Interactions Driving Muscle Regeneration

**Figure 2.**
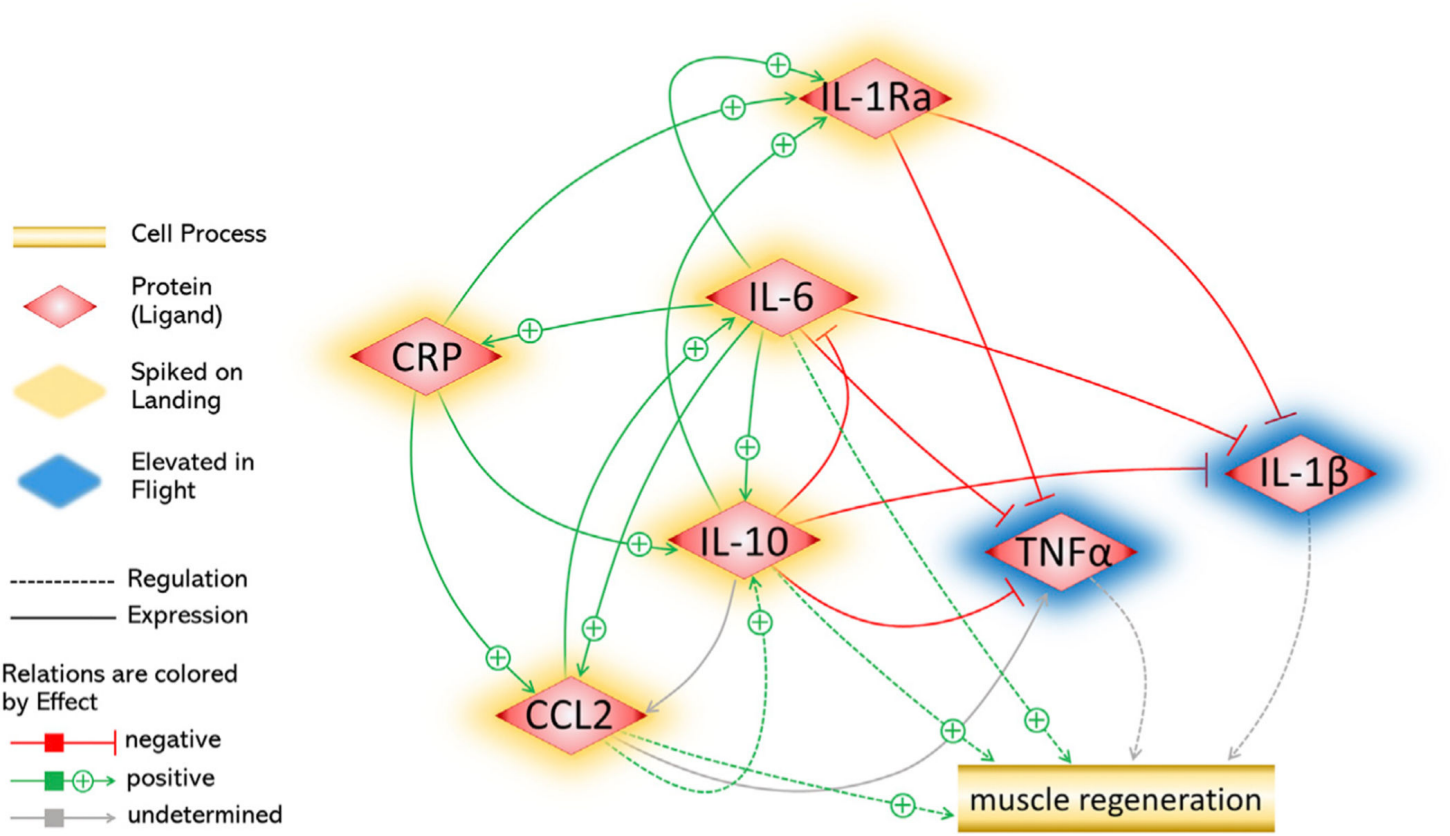
Pathway Interactions Showing the Direct Force Exerted by Each Protein Leading toward Muscle Regeneration Elements that spiked upon landing are highlighted in yellow; those elevated in flight are shown in blue.

The prominent cytokines in the landing signatures map to known mechanistic relationships and biological pathways involved in muscle regeneration (Figure 2). IL-6 is at the center of these different pathways and holds the most connections to the other markers. Of note, IL-6 has two signaling pathways: trans-signaling and classic signaling (Scheller et al., 2011). The former is associated with a pro-inflammatory response (e.g., increases in IL-1β and TNF-α) and the latter is associated with the anti-inflammatory functions of exercise and homeostasis (e.g., inhibitory effect on TNF-α and IL-1) (Chalaris et al., 2010; Scheller et al., 2011; Yan et al., 2016; Del Giudice and Gangestad, 2018). Given this relationship, the data from TW and other astronauts suggest that IL-6 is following a trans-signaling pathway while in flight, since it is elevated along with TNF-α and IL-1(α and β) (ANOVA, *q* < 0.05) (Garrett-Bakelman et al., 2019). However, at R0, IL-6 increased 100%, while TNF-α and IL-1 decreased sharply between R0 and R+5. IL-10 and IL-1ra also increased at R0 (Garrett-Bakelman et al., 2019), as is predicted by the known pathways of muscle regeneration. Taking these combined interactions into consideration, the R0 and R+5 biochemical data from TW suggest that IL-6 mechanism changes upon landing, switching to function through the classical signaling pathway in response to increased muscle loading and gravity.

### In-Flight Marker Shifts

**Figure 3.**
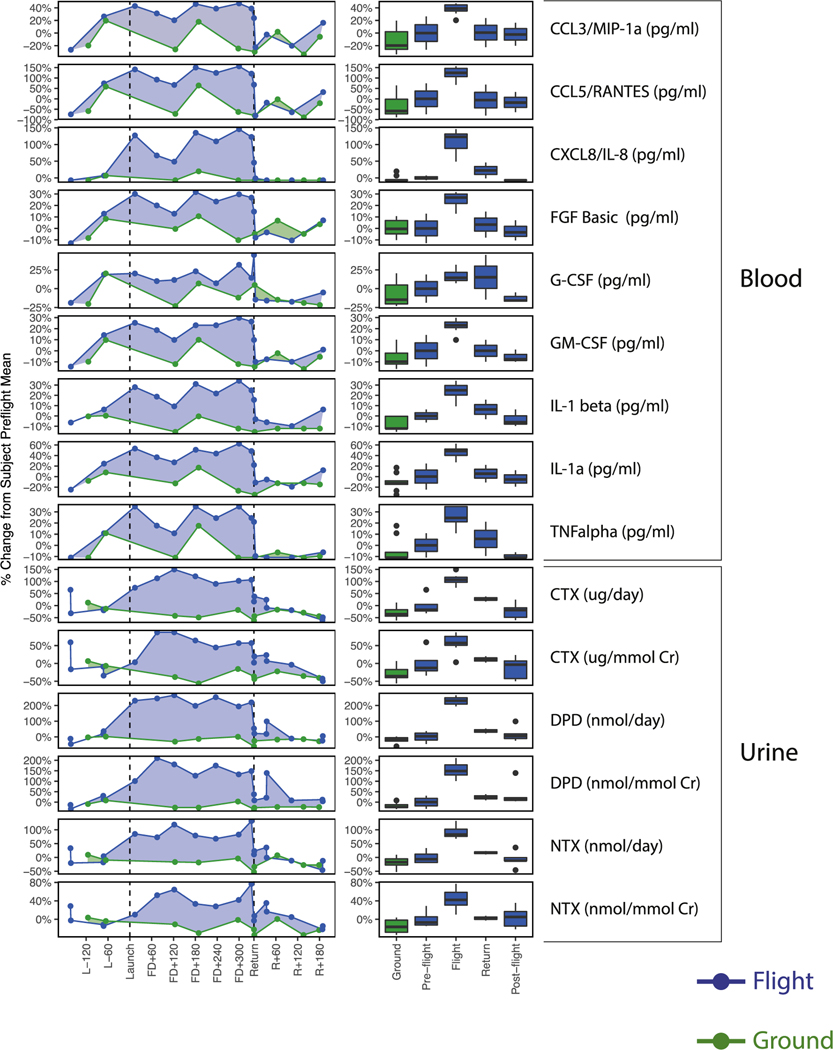
Elevated In-Flight Markers Associated with Bone Metabolism and Early Stages of Muscle Regeneration That Decreased upon Landing For both the line graphs and the boxplots, the y axis is the percent difference from the mean of each subject’s pre-flight value plotted over time. For the line graphs, x axis is number of days during the mission and for the boxplots, the x axis is divided into mission intervals. Blue lines are for TW, and green lines are for HR. See also Figure S1. (Garrett-Bakelman et al., 2019)

A broad range of biochemical markers were elevated during flight and decreased significantly on return (ANOVA, *q* < 0.05; Figure 3) (Garrett-Bakelman et al., 2019). Although many of these are involved in mediating inflammation, cell growth, and cell prolifer ation, as well as tumor proliferation and vascularization (Garrett-Bakelman et al., 2019), they are also involved in musculoskeletal pathways. Most notably, these include inflammatory, osteoclastogenic cytokines (TNF-α, IL-1α, IL-1β, IL-8) (Amarasekara et al., 2018) and chemokines (CCL5, CCL3) (Yano et al., 2005; Vallet et al., 2011), which are involved in osteoclast activation and osteoblast inhibition as well as muscle atrophy. Important mediators include growth factors (fibroblast growth factor [FGF] basic, granulocyte colony stimulating factor [GCSF], granulocyte macrophage colony stimulating factor [GM-CSF]) (Nagaraju et al., 1998; Kuwabara et al., 2001; Park et al., 2007; Yun et al., 2010; Charoenlarp et al., 2017), which are associated not only with processes of muscle differentiation and regeneration but also with osteoclast-mediated bone resorption and inflammation. As with the cytokines elevated in response to landing, the profiles of TNF-α, IL-1β, IL-8, CCL5, and GCSF are similar to those measured in other astronauts (Figure S1). Again, these measured effects are not as pronounced for the astronauts returning from shorter flights compared with TW. This would appear to be a result of these markers not becoming as elevated during the shorter flight. Urinary bone resorption markers that also decreased include urinary deoxypyridinoline (DPD), urinary C-terminal crosslinking telopeptide (CTX), and urinary N-terminal telopeptide (NTX), among others (Garrett-Bakelman et al., 2019). The decrease of these markers immediately following the rise of markers associated with muscle regeneration (Figure 1), further support the suggested interpretation of the landing signatures as a progression from muscular atrophy, inflammation, and bone resorption during flight to muscle regeneration and bone anabolism upon landing.

### Single-Cell Epitope and Expression Analysis

**Figure 4.**
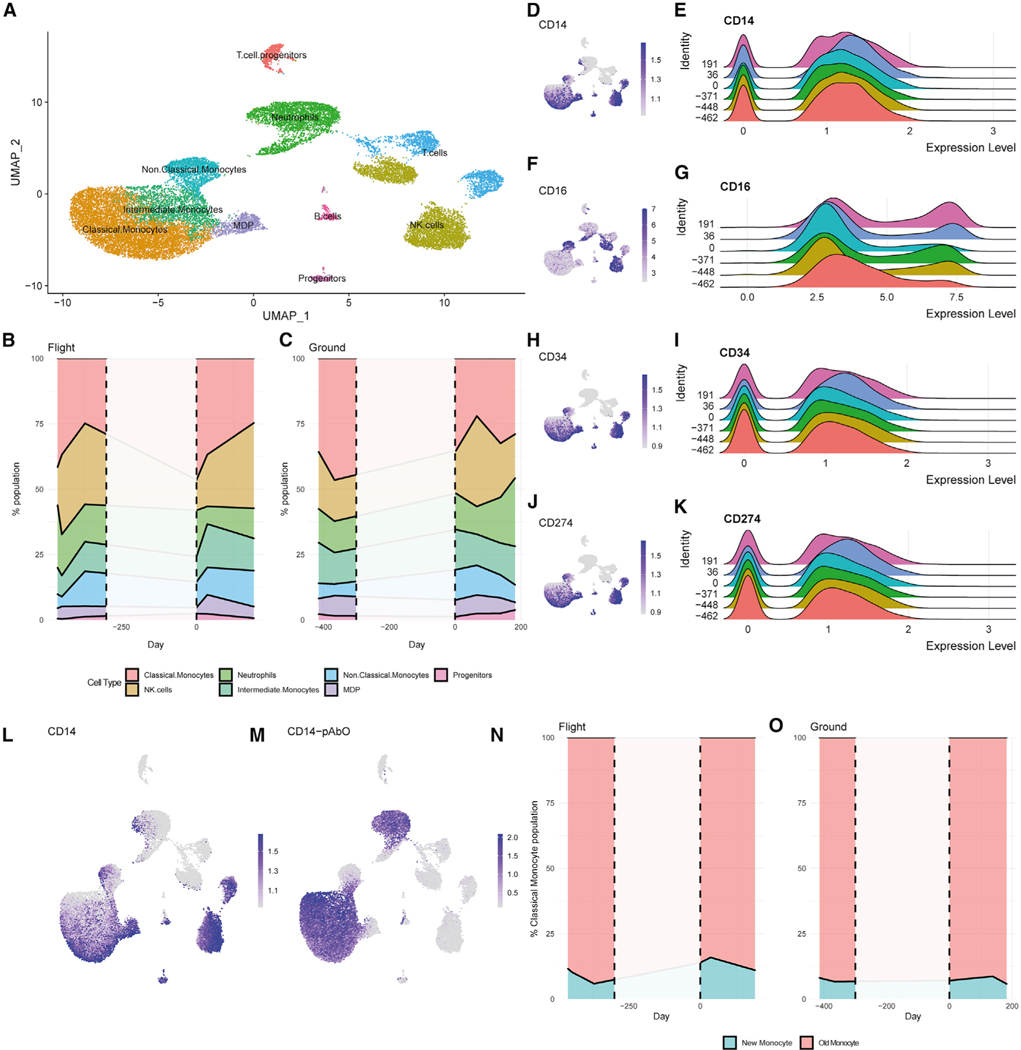
Single-Cell Analysis Shows Cell Population and Expression Consistent with Measured Cytokine Levels (A) Uniform manifold approximation and projection (UMAP) projection of BD Rhapsody single-cell data (n = 23408 after QC) labeled by cell type. (B and C) Stacked line plots showing the percent population based on cluster identification for TW (B) and HR (C). Dotted lines and white space indicate the time that TW was in flight, of which no samples were sequenced for single-cell analysis. (D–K) UMAP and ridge plots showing expression of CD14 (D and E), CD16 (F and G), CD34 (H and I), or CD274 (J and K) from TW. Cells were grouped by day collected relative to R0. (L and M) UMAP showing expression of CD14 mRNA (L) and protein expression through epitope tag (M). (N and O) Stacked line plots showing the percentage of new and old classical monocytes per day for TW (N) and HR (O). Classical monocytes were determined to be new monocytes if CD14 mRNA expression was greater than 1, but CD14 epitope expression was less than 1. See also Figure S2.

Immune cell types were also profiled at the epitope and gene expression levels to discern the impact of spaceflight on cellular composition. Time points include pre-flight, landing, and post-flight data; there is no in-flight data available for single-cell analysis. Clusters were identified through projection of both RNA and epitope expression (Figure 4A; Figure S2). Composition analysis was done after removal of T and B cell populations, as the samples were CD4, CD8, and CD19 depleted (Figures 4B and 4C). Although the composition of immune cell populations seemed to be impacted both on landing and during post-landing recovery, these values were not out of the range observed in the matching ground samples. The monocyte population seemed to be most affected in terms of composition, with a large increase in the number of classical monocytes compared with the other profiled cell populations on the day TW returned to Earth. This is supported by the number of cells at day R0 expressing high levels CD14 compared with CD16 (Figures 4D–4G). By days 36 and 191 post-flight, the numbers of classical monocytes decreased back to levels similar to the measured pre-flight values, and the ratio of CD14 and CD16 high expressing cells is more similar to the pre-flight time points. By contrast, the circulating monocyte-dendritic cell precursor (MDP) and progenitor populations do increase post-flight; this is supported by an increase in the expression of CD34 (Figures 4H and 4I). There was also an increase in CD274, a marker of monocyte activation, at days 36 and 191 post-flight (Figures 4J and 4K). Additionally, the expression of CD14 mRNA and CD14 epitope was compared to determine which cells were likely new classical monocytes. Cells were defined to be new classical monocytes if their scaled CD14 mRNA expression was greater than 1, while their scaled CD14 epitope expression was less than 1 (Figures 4L and 4M). The proportion of new classical monocytes to the total monocyte population was then determined for each time point (Figures 4N and 4O). The increase in new monocytes in the post-flight time points is consistent with the increase in circulating MDP and progenitor cells.

**Figure 5.**
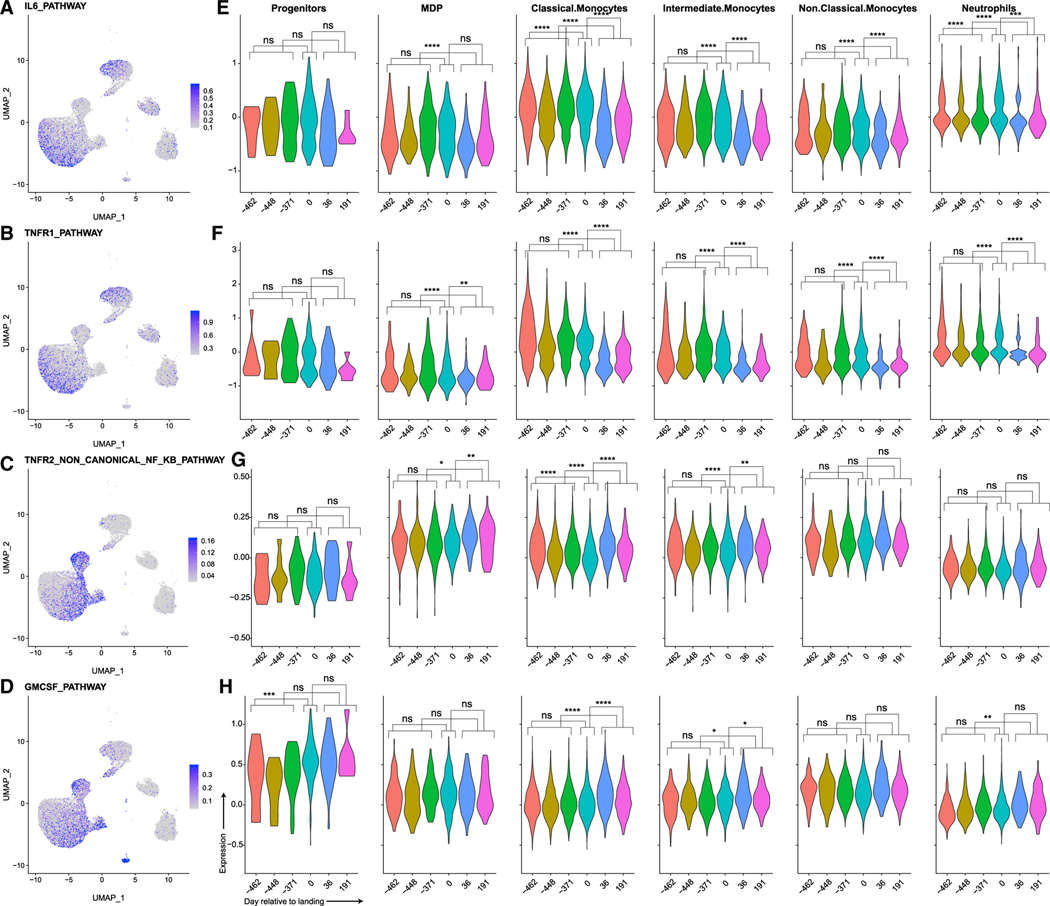
Expression of Pathways Associated with Initiation and Closure of Inflammation and Skeletal Muscle Regeneration in the Monocyte Subpopulations and Neutrophils Show the Effects of Landing on Specific Populations (A–D) UMAP showing the expression of the Biocarta IL-6 pathway (A), Biocarta TNFR1 pathway (B), Reactome TNFR2 non-canonical NF-κB pathway (C), and primary immunodeficiency (PID) GM-CSF pathway (D). (E–H) Violin plots showing the average expression of these pathways in progenitors, MDP, classical monocytes, intermediate monocytes, non-classical monocytes, and neutrophil cells, separated by day relative to landing. See also Table S1.

In addition to the holistic composition analysis, we determined expression of select genes in pathways related to the measured biochemical response to landing in the monocyte-related population at each time point, including IL-6, TNF receptor 1 (TNFR1), TNFR2, non-canonical nuclear factor κB (NF-κB), and GM-CSF (Figure 5). Cells were grouped by day into pre-flight (where L is days before launch, L-462, L-448, L-414, L-371 for TW and GD-366 for HR), landing (where R us days post return, R0 for TW and GD+3 for HR), and post-flight (R+36, R+191 for TW and GD+66 for HR) and tested to determine whether the changes in expression were significant for these signatures by Wilcoxon rank-sum test in a pairwise fashion (Table S2). IL-6 pathways are most affected in the classical monocyte population, where we see statistically significant differences (all adjusted p values < 9.33E–14) between pre-flight, landing, and post-flight expression (Figure 5E; Table S2), with an increase at R0 compared with pre-flight and subsequent decrease post-flight. MDPs and intermediate and non-classical monocytes also show significant differences between landing and post-flight measurements as well as between pre-flight and post-flight, with decreases in post-flight values compared with landing and pre-flight (Table S2).

The TNFR1 and TNFR2 non-canonical NF-κB pathways were also affected. TNFR1 showed statistically significant differences between landing and post-flight measurements for MDPs and classical, intermediate, and non-classical monocytes, with decreases in post-landing values compared with landing and pre-flight values (all *q* < 0.01) (Figures 5F and 5G; Table S2). Similar to IL-6, TNFR2 was also most affected in its classical monocyte population, although appearing to have an almost inverse relationship with IL-6 in its pre-flight, landing, and post-flight responses. TNFR2 shows a decrease in expression at R0 compared to pre-flight and subsequent increase post-flight. MDPs and intermediate monocytes also show significant differences between landing and post-flight measurements, as well as between pre-flight and post-flight measurements, with increases in post-flight values compared with landing and pre-flight values.

Given the rapid dynamics of the MDPs and other immune precursor populations, we then compared the response to the GM-CSF-associated cells between the pre-flight and landing samples. These data showed that the only population with a significant increase in expression is in the progenitor cells (Figure 5H; Table S2). By contrast, when comparing the post-flight samples to either the pre-flight or landing samples, we see significant increases in expression within the classical and intermediate monocytes, as well as the neutrophils. The post-flight progenitor cells also had significantly increased expression compared with the pre-flight samples, but not the landing samples. The increase in GM-CSF response in the circulating progenitor cells at R0 correlates with the increase in their proportion of PBMCs. At R+36 and R+191, the classical and intermediate monocytes had elevated levels of GM-CSF pathway genes. This indicates a general expansion of the monocyte population, as evidenced by the increase in the new classical monocyte population. GM-CSF alone does not influence polarization of the monocytes, which is evidenced by an increase in new classical monocytes being accompanied by a decrease in the ratio of classical-to-non-classical monocytes, consistent with initiation of skeletal muscle repair (Wicks and Roberts, 2016; Kim et al., 2018).

## DISCUSSION

### Cytokine Shifts Mediating Inflammation, Muscle Regeneration, and Bone Anabolism

The biochemical profiling and single-cell analysis taken together suggest mechanisms involved in a physiological response to muscle regeneration and bone anabolism on landing. Muscle unloading conditions occurred concomitantly with the elevation of biomarkers associated with bone resorption, muscle atrophy, and regeneration during flight in TW. Soon after returning to 1g, muscle activation likely initiated cascades driven by the large increase of muscle-derived IL-6, comparable with signal cascades involving the same pathways and cytokines in studies involving intense exercise. While there are many factors influencing the changes in expression of the measured cytokines, muscle is the largest internal organ with autocrine, paracrine, and endocrine functions and is therefore most likely to be among the greatest contributors to the biochemical changes observed in TW (Pedersen, 2009, 2013; Hamrick, 2011).

It is well established that muscle and bone mass are closely linked across growth, development, and aging on Earth. Spaceflight has been shown to cause skeletal muscle atrophy as a result of mechanical unloading, in spite of heavy resistive exercise regimens (Fitts et al., 2000; Brooks and Myburgh, 2014). Ground-based studies have also demonstrated that muscle atrophy is associated with bone loss and conversely, mechanical stimuli from muscle with bone anabolism and osteogenesis (Hamrick, 2011). These ground studies suggest that an increase of bone resorption markers in the flight data may be linked to muscle unloading (Ko et al., 2020) and atrophy, although more flight studies would be needed. The elevated in-flight markers include osteoclastogenic cytokines TNF-α, IL-1β, and IL-1α, among others, all of which also drive muscle wasting in chronic inflammatory conditions (Yang and Hu, 2018). Moreover, all the osteoclastogenic cytokines and chemokines, as well as the elevated bone resorption markers shown in Figure 3, decrease significantly immediately after landing, following an ~100-fold increase in IL-6, again likely due to increased muscle loading. Noting that although loads such as standing and walking may not have been high, they were novel in comparison with spaceflight, both by using muscles not applied during exercise countermeasures and by having a higher cumulative load throughout the day.

### Parallels between Pathogenic Immune Response and Muscle Regeneration

Under normal weight-bearing conditions, muscle regeneration during acute injury due to exercise can be divided into three major time-dependent phases that parallel an immune response to pathogens: the first and second phases are pro-inflammatory and the third phase is anti-inflammatory (Yang and Hu, 2018). In the first phase, pro-inflammatory cytokines including TNF-α and IL-1 are released to promote requisite inflammation to the affected muscle tissue (Gordon and Galli, 1990; Gorospe et al., 1996; Radley and Grounds, 2006). The second phase involves the conversion of monocytes to classically activated, pro-inflammatory M1 macrophages and the recruitment of T cells, which also express and maintain large amounts of TNF-α and IL-1β. The third phase is marked by the switch from pro-inflammatory M1-type to anti-inflammatory M2-type macrophages, which produce IL-10 to suppress the local inflammatory response and promote regeneration (Stout and Suttles, 2004; Yang and Hu, 2018). In the case of atrophy from non-use, however, mouse studies have indicated that unloading conditions such as spaceflight can inhibit or delay skeletal muscle regeneration by interfering with the recruitment of macrophages and abnormally persistent neutrophils (Kohno et al., 2012). The phenotype is thus predominantly driven by pro-inflammatory M1-type macrophages that are high in expression of TNF-α and IL-1β, leading to prolonged inflammation and incomplete muscle regeneration (Kohno et al., 2012).

The elevated in-flight levels of TNF-α, IL-1(β and α), along with the lack of IL-10 elevation in the biochemical data, suggest that while all three stages of muscle regeneration may be present during flight as a result of daily exercise, the first two pro-inflammatory stages appear to predominate over the third anti-inflammatory stage. On landing, the regeneration process progresses to the final, anti-inflammatory phase of regeneration as is supported by the increase of IL-10 and IL-1ra as well as the simultaneous decrease of TNF-α and IL-1(β and α).

### Shifts in Monocyte Populations and Select Gene Pathways Suggest Muscle Regeneration

The single-cell data show a significant increase in the classical monocyte population at R0 compared with the pre-flight samples. Since there are no in-flight data available, it is unknown whether this population was elevated prior to landing. However, this might be the case as this would be consistent with chronic inflammation. Moreover, the change observed in the post-landing single-cell data, showing a progressive decrease in the classical monocyte population (M1 progenitor) and a progressive increase in non-classical (M2 progenitor) monocytes, supports the biochemical data that suggest a disrupted regeneration process in-flight. The decline in the post-landing neutrophil population may be indicative of change in the state of persistence in muscle, or re-acclimation to the terrestrial environment (and microbiome) (Singh et al., 2018), but it is difficult to say without knowing inflight values (Castro-Wallace et al., 2017; McIntyre et al., 2019). Expression of select gene pathways further supports this interpretation, particularly in the apparent near-inverse relationship between IL-6 and TNFR2, mainly in the classical monocyte population. It is also interesting to note the R0 values in the single-cell data returned to pre-flight values by R+36.

### Pharmacological Interventions

Insights from the multi-omics data generated in the NASA Twins Study and the comprehensive biochemical profiling analyzed can potentially translate into targeted pharmacotherapies for astronauts to ameliorate symptoms experienced on return to Earth. However, these must be weighed carefully, as many potential interventions would impair muscle regeneration. Here, we review some of the pharmacological measures specific to different cytokines and chemokines with significant shifts in TW’s return, which are also summarized in Table 1.

Of the five cytokines that spiked on landing, only two appear to be good candidates for pharmacological intervention: IL-6 and IL-1ra. IL-6 is at the center of the pathways involving muscle regeneration, which would make it an ideal target for pharmacological manipulation. However, global inhibition of IL-6 can result in impaired muscle growth (Grivennikov et al., 2009; Scheller et al., 2011; Hoene et al., 2013; Muñoz-Cánoves et al., 2013). Yet, selective inhibition of IL-6 trans-signaling is possible using soluble gp130 (sgp130) to bind the IL-6/soluble IL-R6 (sIL-6R) complex in circulation (Kaiser et al., 2018). Moreover, studies suggest that classic signaling may mediate beneficial effects on bone repair and that selective inhibition of IL-6 trans-signaling may have therapeutic potential (Grivennikov et al., 2009; Scheller et al., 2011; Kaiser et al., 2018). As opposed to selective inhibition of trans-signaling, in-flight enhancement of IL-6 is another possibility. Although IL-6 is already slightly elevated in-flight, the source of IL-6 is dictating an inflammatory, trans-signaling pathway as is supported by the elevated levels of TNF-α and IL-1. In studies of IL-6 enhancement, recombinant human IL-6 (rhIL-6) infusion inhibited levels of TNF-α similarly to the effects of exercise, or increased production of IL-1ra and IL-10 without increasing TNF-α (Starkie et al., 2003; Steensberg et al., 2013). These provide potential options for control of the inflammatory downstream effects of IL-6 trans-signaling while in flight, although not necessarily on landing since there is already an abundance of muscle-derived IL-6. Enhancement of IL-1ra may be a strategy better suited for landing. IL-1ra is involved in the resolution of inflammation in skeletal muscle repair after exercise by blocking IL-1 inflammatory pathways as well as IL-6 trans-signaling without affecting muscle-generated IL-6 (Pedersen et al., 2001; Nordmann et al., 2015). A clinical trial by Nordmann et al. (2015) found that IL-1 antagonism with a recombinant, non-glycosylated form of the human IL-1ra (anakinra) improved glycemia and decreased systemic inflammation including trans-signaling of IL-6 without affecting muscle-derived IL-6, which is independent of IL-1 signaling.

Inhibition of CRP on landing may be unfavorable for muscle regeneration and must be considered carefully. Like IL-6, CRP has pro- and anti-inflammatory roles in many processes related to inflammation and tissue maintenance (Del Giudice and Gangestad, 2018). In the absence of inflammation, CRP production is stimulated by IL-6 via classic signaling. Through this pathway, CRP is acting in an anti-inflammatory role by stimulating the release of IL-10 and IL-1ra, contributing to tissue repair and clearance of damaged cells, as well as resolving inflammation by inducing the anti-inflammatory M2 phenotype in macrophages, which promotes tissue repair and wound healing (Del Giudice and Gangestad, 2018).

Although CCL2 is often a pharmacological target for inflammation, it plays an essential role inducing a requisite inflammatory response to repair and regenerate skeletal muscle (Deshmane et al., 2009; Lu et al., 2011). Human clinical trials in most investigational drugs that target CCL2/CCR2 signaling have failed except for plozalizumab (Gilbert et al., 2011). Alprazolam, a benzodiazepine medication, has been found to inhibit IL-1α-elicited CCL2 production without inhibiting IL-8 production (Oda et al., 2002). However, in the case of returning astronauts, any attempts to reduce CCL2 production or reduce CCR2 expression would need to be carefully weighed, since poor muscle regeneration may be a significant side effect of this line of therapy and would not be warranted to treat an acute and temporary elevation of CCL2 (Lu et al., 2011).

Enhancement of IL-10 would initially seem a plausible avenue, since IL-10 plays a central role in regulating the switch of macrophages from the inflammatory M1 to the anti-inflammatory M2 phenotype in injured muscle, as well as inhibiting the production of inflammatory IL-1α, IL-1β, GCSF, and GM-CSF (Deng et al., 2012; Londhe and Guttridge, 2015). However, clinical studies find that IL-10 supplementation mainly benefits subjects who have endogenously lower expression of IL-10 and who have active disease states (Marlow et al., 2013). Moreover, although IL-10 is required for normal growth and regeneration of injured muscle, there appears to be a dose-dependent effect, and supraphysiological levels may slow the growth of regenerative fibers (Lauw et al., 2000; Perdiguero et al., 2011; Deng et al., 2012). Therefore, use of IL-10 must also be carefully considered.

In conclusion, these data indicate that the severity of the human body’s response to returning to gravity is somewhat dependent on the length of time an astronaut spends in space. While these findings are striking, and the suggested countermeasures plausible, it must be cautioned that these are results of an n = 2 study, plus a small confirmatory cohort of 28 astronauts. More data for longer missions need to be collected to prepare for long-duration Martian missions (Nangle et al., 2020), including new, dynamic measures from blood and serum such as cell-free nucleic acids and exomes (Bezdan, 2020), miRNAs (Malkani, 2020), telomere dynamics (Luxton, 2020), and mitochondrial stress (Silveira, 2020). Also, while some of these biochemical and genetic signatures are recurrent, not every astronaut will exhibit the same biochemical profile shifts or respond the same way to the same medical interventions (Vernice et al., 2020). Indeed, such variability is the basis for the field of pharmacogenomics, and personalized approaches to aerospace medicine are now emerging (Iosim et al., 2019; Schmidt et al., 2020). This catalog of biomarkers, their targets, and methods for pharmacological manipulation can help guide future work and understanding of the body’s adaptations to “normal” gravity when returning to Earth (1g), or eventually the less strenuous gravity of the moon (16%) or Mars (38%).

## STAR★METHODS

### RESOURCE AVAILABILITY

#### Lead contact

Further information and requests for resources and reagents should be directed to and will be fulfilled by the Lead Contact, Christopher Mason (chm2042@med.cornell.edu)

#### Materials availability

This study did not generate any new unique reagents.

#### Data and code availability

The NASA Life Sciences Data Archive (LSDA) is the repository for all human and animal research data, including that associated with this study. LSDA has a public facing portal where data requests can be initiated (https://lsda.jsc.nasa.gov/Request/dataRequestFAQ). The LSDA team provides the appropriate processes, tools, and secure infrastructure for archival of experimental data and dissemination while complying with applicable rules, regulations, policies, and procedures governing the management and archival of sensitive data and information. The LSDA team enables data and information dissemination to the public or to authorized personnel either by providing public access to information or via an approved request process for information and data from the LSDA in accordance with NASA Human Research Program and Johnson Space Center (JSC) Institutional Review Board direction.

### EXPERIMENTAL MODELS AND SUBJECT DETAILS

#### NASA Twins Study sample collection

A pair of monozygotic twins were studied for 25 months, during which one subject (Flight subject, TW) spent 340 days aboard the International Space Station (ISS) while his identical twin (Ground subject, HR) remained on Earth (Garrett-Bakelman et al., 2019). Subjects were male and aged 50 at the beginning of the study. Both subjects had different amounts spaceflight exposure prior to the study (Flight subject = 180 days total, Ground subject = 54 days total). Multiple blood samples were collected (N_flight_ = 19, N_ground_ = 12) from both subjects beginning approximately 6 months prior to the launch date, during the 340 days aboard the ISS, and 6 months after return (Garrett-Bakelman et al., 2019).

Blood and urine samples were collected as previously described (Smith et al., 2012b, 2015; Garrett-Bakelman et al., 2019). Blood sample collection, sorting, RNA extraction, RNA sequencing analysis, quality control, library preparation, and sequencing were previously described (Garrett-Bakelman et al., 2019). Briefly, for lymphocyte analyses, samples were either mixed by inversion, subjected to mononuclear cell separation to isolate PBMCs and immediately frozen at −80°C (for both Earth and ISS collections), or were freshly collected and immediately processed. Fresh samples collected aboard the ISS were returned to Earth in the Soyuz capsule and kept at 4°C until processing (approximately 35 h from collection to processing). CD4, CD8, and CD19 cells were isolated from fresh samples and the remaining Lymphocyte-depleted fraction (LD) was retrieved from sequential magnetic bead positive selections. Frozen samples were processed to retrieve the PBMCs from the CPT vacutainers.

### METHOD DETAILS

#### Proteomics on NASA Twins Study samples

Data generation was described in Garrett-Bakelman et al., 2019.

#### Targeted urine proteomics

An LC-MS/MS based targeted proteomic approach was applied to assess 24 h pooled urine samples targeting a panel of 20 proteins which are found detectable in urine samples derived from healthy subjects and individuals with pathological conditions: podocalyxin (PODXL), renin receptor (RENR), urokinase-type plasminogen activator (UROK), pro-epidermal growth factor (EGF), Collagen alpha-1(I) chain (CO1A1), Collagen alpha-1(III) chain (CO3A1), Intercellular adhesion molecule 1 (ICAM1), Cathepsin D (CATD), Matrilysin (MMP7), Insulin-like growth factor-binding protein 3 (IBP3), Insulin-like growth factor-binding protein 2 (IBP2), Vascular cell adhesion protein 1 (VCAM1), Connective tissue growth factor (CTGF), Syndecan-4 (SDC4), Aquaporin-2 (AQP2), Selenoprotein P (SEPP1), Urokinase plasminogen activator surface receptor (UPAR), Insulin-like growth factor-binding protein 7 (IBP7), Endothelial cell-selective adhesion molecule (ESAM), Cytosolic non-specific dipeptidase (CNDP2). Urine (0.2 mL) was supplemented with 1 μg of α-amylase from *Aspergillus oryzea*, which helps provide bulk protein to optimize protein precipitation and helps control for the variability in digestion from sample to sample. Ice-cold acidified methanol:acetone 50%:50% (v:v, 0.8 mL) was used to precipitate proteins for 24 h at −20°C. Precipitated proteins are dried under vacuum and then reconstituted in 0.5% sodium deoxycholate in 50 mM ammonium bicarbonate. Proteins were reduced in 5 mM dithiothreitol for 1 h at 60°C, alkylated in 15 mM iodoacetamide for 30 min at room temperature in the dark, and then digested with 1 μg trypsin (Worthington Biomedical, Lakewood, NJ) for 2 h at 37°C with shaking (Thermomixer, 1400 rpm). Deoxycholate was precipitated by the addition of HCl (final concentration of 200 mM), which was removed by centrifugation. The supernatant was desalted using solid phase extraction (HLB mElute plate, Waters, Milford, MA). The eluted peptides were dried under vacuum and reconstituted in 5% acetonitrile in 0.1% formic acid, which was then spiked with internal standard peptides. Proteins of interest were quantified using LC-MS/MS on a Thermo Orbitrap mass spectrometer coupled to an Easy-nLC liquid chromatography system (Thermo Scientific, Waltham, MA). Selected peptides from the proteins of interest were monitored by selecting their precursor ions in the isolation quadrupole (selection window 1.5 Da) and full scan MS/ MS after HCD fragmentation (NCE 27) in the Orbitrap analyzer using the lowest resolution (17,500) setting to increase scan rate. Scheduled acquisition with five-minute acquisition windows were set up for each peptide precursor using Skyline software (MacLean et al., 2010), allowing a maximum of 30 concurrent PRM experiments at any given time. Acquired data were then processed in Skyline, and automated integration was 10 manually checked for each peptide chromatogram.

#### Untargeted urine proteomics

The digested peptides (375 ng) were injected on a trap column (40 × 0.1 mm, Reprosil-Pur 120 C18-AQ, 5 um, Dr.Maisch GmbH, Germany), desalted for 5 min at a flow of 4 μL/min and separated on a pulled tip analytical column (250 × 0.075 mm, Reprosil-Pur 120 C18-AQ, 5 um, Dr. Maisch GmbH, Germany) with a three-segment linear gradient of acetonitrile, 0.1%FA (B) in water, 0.1%FA (A) as follows: 0–3 min 1%–7%B, 3–53 min 7%–25%B, 53–60 min 25%–35%B followed by column wash at 80%B and re-equilibration at a flow rate 0.4 uL/min (Waters NanoACQUITY UPLC). Analysis using the data independent acquisition was performed in two steps. A chromatogram library, peptide and, protein identification, and spectral library was performed with narrow mass window selection for the MS/MS (2 m/z) on a pooled sample. The study samples were analyzed with a wider mass window selection for MS/MS (10 m/z). The data independent analysis was performed on Orbitrap Fusion Lumos (Thermo Scientific) as follows: single full scan MS1 is acquired over m/z range 395–1005 at resolution 120,000 followed by 60 MS/MS spectra with a 10 m/z window selection stepped over the range 400–1000 with HCD fragmentation mode (NCE 30) and MS/MS acquisition in the Orbitrap analyzer at resolution 17,500. For Chromatogram library generation a sample pooled across multiple subjects and time points was analyzed in a similar fashion in 6 separate injections with the DIA acquisition using 2 m/z window over m/z ranges 400–520, 520–640, 640–760, 760880, 880–1000.

#### Untargeted plasma proteomics

Plasma proteins were reduced by 5 mM tris (2-carboxyethyl) phosphine and alkylated using 10 mM iodoacetamide. They were then digested by trypsin using 1:20 protein ratio. Tryptic peptides of plasma samples were separated on a NanoLC 425 System (SCIEX). 5 ul/min flow was used with trap-elute setting using a 0.5 × 10 mm ChromXP (SCIEX). LC gradient was set to a 43-minute gradient from 4%–32% B with 1 h total run. Mobile phase A was 100% water with 0.1% formic acid. Mobile phase B was 100% acetonitrile with 0.1% formic acid. 8 ug load of undepleted plasma on 15 cm ChromXP column.

#### Cytokine assays

Levels of circulating cytokines in the blood were measured using a 63-plex Luminex antibody-conjugated bead capture assay (Affymetrix) that has been extensively characterized and benchmarked by the Stanford Human Immune Monitoring Center (HIMC). Human 63-plex kits were purchased from eBiosciences/Affymetrix and used according to the manufacturer’s recommendations with modifications as described below. Briefly, beads were added to a 96-well plate and washed using a Biotek ELx405 washer. Samples were added to the plate containing the mixed antibody-linked beads and incubated at room temperature for 1 h followed by overnight incubation at 4°C with shaking. Cold and room temperature incubation steps were performed on an orbital shaker at 500–600 rpm. Following the overnight incubation, plates were washed using a Biotek ELx405 washer and then biotinylated detection antibody added for 75 min at room temperature with shaking. The plate was washed as describe earlier and streptavidin-PE was added. After incubation for 30 min at room temperature, a wash was performed as above and reading buffer was added to the wells. Each sample was measured in duplicate. Plates were read using a Luminex 200 instrument with a lower bound of 50 beads per sample per cytokine. Custom assay control beads by Radix Biosolutions were added to all wells.

#### Single cell RNA and epitope sequencing on NASA Twins Study samples

Single-cell BD Rhapsody data were generated according to the BD Rhapsody Express Single-Cell Analysis System Instrument User Guide. Briefly, single cell suspensions were generated from the Leukocyte Depleted (CD4, CD8 and CD19 depleted) fraction of cells previously described (Garrett-Bakelman et al., 2019). Cells from each sample were labeled with sample tags, pooled, and washed three times with FACS buffer. Cells were then stained with BD AbSeq Ab-Oligo reagents, washed twice and resuspended at approximately 20,000 cells in 620 μL. Stained and washed cells were isolated using the BD Rhapsody Express Single-Cell Analysis System using the manufacturer’s protocol (BD Biosciences). Cells were loaded across three BD Rhapsody nanowell cartridges. These cartridges were loaded with Cell Capture Beads (BD Biosciences), then incubated at room temperature for 15 s on a shaker set to 1,000 rpm. Cells were lysed and cell capture beads were retrieved, washed, treated with Exonuclease I and reverse transcriptase. Targeted amplification of cDNA with Human Immune Response Panel primers and custom supplemental panel was done through 10 PCR cycles. Custom gene and epitope panel designed with help from BD (see Table S1 for probe list). PCR products were purified, with mRNA and AbSeq PCR products being separated by double-sided selection SPRI select beads. mRNA was further amplified with 15 more PCR cycles. Final libraries were indexed with 8 PCR cycles. A Bioanalyzer (D1000 HS, Agilent) was used to assess library quality and a Qubit dsDNA HS Kit (ThermoFisher, #Q32854) and Qubit Fluorometer was used to determine library DNA concentration. Libraries were diluted to 2nM, multiplexed, and sequenced on three NovaSeq-6000 lanes. Mean read depth per cell was 13,244.31 mRNA reads per cell and 11,506.98 AbSeq reads per cell for a combined total of 24,751.30 reads per cell.

### QUANTIFICATION AND STATISTICAL ANALYSIS

#### Analysis of Proteomics from NASA Twins Study Samples

Biochemical data were processed as described in Garrett-Bakelman et al., 2019. Samples were normalized to pre-flight mean for each astronaut.

#### Targeted urine proteomics

Acquired data were then processed in Skyline and automated integration is manually checked for each peptide chromatogram.

#### Untargeted urine proteomics

A spectral library of identified peptides and proteins was generated using a pipeline combining two approaches – a combination of DIA Umpire to extract MS/MS spectra followed by a Comet database search, and a direct approach using PECAN (Ting et al., 2017) lemented in EncyclopeDIA (Searle et al., 2018) Proteins and peptides were then quantified in each sample using EncyclopeDIA and Skyline (MacLean et al., 2010; Searle et al., 2018) using with an mProphet probability scoring algorithm (http://mprophet.org) and are filtered at q-value = 0.01 to generate list of quantifiable peptides and proteins together with corresponding quantitative responses. The data was further processed and quantified in Skyline and mapDIA (Teo et al., 2015) software packages. Individual protein and peptide responses were exported for further statistical analysis. Peak areas for each endogenous peptide were normalized to internal standard peptides from a stable isotope-labeled internal α-amylase protein standard to generate a peak area ratio for each peptide in each sample. Peak area ratios for each protein were averaged, and protein peak area ratios then normalized to protein peak area ratios for calibrator samples included in each digestion batch. The resulting calibrated protein peak area ratios were used as relative concentrations of each protein.

#### Untargeted plasma proteomics

MS analyses were performed using SWATH®Acquisition on a TripleTOF®6600 System equipped with a DuoSpraySource and 25 μm I.D. electrode (SCIEX). Variable Q1 window SWATH Acquisition methods (100 windows) were built in high sensitivity MS/MS mode with Analyst®TF Software 1.7. Peakgroups from individual runs were statistically scored with pyProphet and all runs were aligned using TRIC to produce a final data matrix. Protein abundances were computed as the sum of the three most abundant peptides (top3 method). After log-transformation and scaling by median count, a linear model was fit to the data. The false discovery rates (FDRs) were estimated using the “p.adjust” function in R. Proteins were considered discriminant when the adjusted p value was below 0.05.

#### Cytokine assays

After log-transformation and scaling by median count, linear models were fit to the data. The false discovery rates (FDRs) were estimated using the “p.adjust” function in R. Cytokines were considered discriminant when the adjusted p value was below 0.05.

#### Analysis of scRNA and epitope sequencing from NASA Twins Study Samples

Fastq files were uploaded to Seven Bridges Genomics. Data were demultiplexed and sequences analyzed with BD’s Rhapsody pipeline (BD Rhapsody Analysis Pipeline 1.4 Beta) on Seven Bridges (https://www.sevenbridges.com/). This generated a sparse matrix file of features by barcodes. This sparse matrix data were then read into R using the R package Seurat 3.2.0 (Stuart et al., 2019), and standard quality control was run to remove cells with few genes or likely doublets. Data were then scaled and normalized. Linear dimensional reduction was performed by calculation of PCA from the most variable genes. Cells were then clustered using a resolution value of 0.5 and visualized by UMAP. Module scores were calculated using the AddModuleScore function with a control value of 5. Individual genes and module scores were projected and used to identify appropriate classification of clusters. Clusters were assigned to cell populations based on gene markers. Significance in module scores between cell populations calculated by Wilcoxon rank sum and corrected by Benjamini Hochberg (5% FDR).

## Supplementary Material

1

2

## Figures and Tables

**Table 1.** Known Drugs That Target Levels of Markers Associated with Initiation or Closure of Inflammation

Target	Drug Class	Name	Comment

IL-6	anti-IL-6	sgp130	mouse studies on bone only
	recombinant human protein	rhIL-6	human study in muscle
CRP	cyclooxygenase inhibitors	aspirin	effective in serum
		rofecoxib	effective in serum
		celecoxib	effective in serum
	platelet aggregation inhibitors	clopidogrel	effective in serum
		abciximab	effective in serum
	lipid lowering agents	statins	effective in serum
		ezetimibe	effective in serum
		fenofibrate	effective in serum
		niacin	effective in serum
	beta-adrenoreceptor antagonists and antioxidants	vitamin E	effective in serum
	ACE inhibitors	ramipril	effective in serum
		captopril	effective in serum
		fosinopril	effective in serum
		enalapril	not effective
		trandolapril	not effective
	ARBs	valsartan	effective in serum
		irbesartan	effective in serum
		olmesartan	effective in serum
		telmisartan	effective in serum
		losartan	inconsistent results
		candesartan	inconsistent results
	antidiabetic agents	rosiglitazone	effective in serum
		pioglitazone	effective in serum
	calcium channel antagonists		inconsistent results
	miscellaneous	hydro chlorothiazide	not effective
		oral estrogen	not effective
		vitamin C	not effective
TSH	hormone	levothyroxine	not suitable for transient hypothyroidism
CCL2/MCP1	CCR2 antagonist	plozalizumab (MLN1202)	investigational drug not tested in muscle
	benzo diazepine	alprazolam	no human studies in muscle
IL-10	recombinant human protein/anti-inflammatory	Tenovil (rhuIL-10)	mouse study in muscle
IL-1ra	recombinant human protein/interleukin-1 inhibitors	Kineret (rhIL-1ra)	clinical trial in muscle

Citations include Oda et al. (2002), Starkie et al. (2003), Prasad (2006), Gilbert et al. (2011), Rugge et al. (2011), Deng et al. (2012), Steensberg et al. (2013), Nordmann et al. (2015), and Kaiser et al. (2018). Abbreviations are as follows: ACE, angiotensin-converting enzyme; ARB, angiotensin receptor blocker; TSH, thyroid-stimulating hormone.

**Table:** KEY RESOURCES TABLE

REAGENT or RESOURCE	SOURCE	IDENTIFIER

Antibodies		

Bacterial and Virus Strains		

Biological Samples		

NASA Twins Study Lymphocyte Depleted Cells	Consented Donors	N/A
NASA Twins Study Plasma	Consented Donors	N/A
NASA Twins Study Urine	Consented Donors	N/A
NASA Plasma	Consented Donors	N/A

Chemicals, Peptides, and Recombinant Proteins		

Critical Commercial Assays		

Rhapsody AbSeq reagent pack	BD Biosciences	Cat#633771; RRID: AB_2870297
Rhapsody Human Immune Response panel	BD Biosciences	Cat#633750; RRID: AB_2870293
Rhapsody custom gene panel (See Table S1)	BD Biosciences	Cat#633743; RRID: AB_2870292
Human Single cell multiplexing kit	BD Biosciences	Cat#633781; RRID: AB_2870299

Deposited Data		

NASA Twins Study BD Rhapsody Data	This paper and Malkani, 2020	LSDA (NASA)https://lsda.jsc.nasa.gov/Request/dataRequestFAQ
NASA Twins Study Cytokine Results	Garrett-Bakelman et al., 2019	LSDA (NASA)https://lsda.jsc.nasa.gov/Request/dataRequestFAQ
NASA Twins Study Metabolite Results	Garrett-Bakelman et al., 2019	LSDA (NASA)https://lsda.jsc.nasa.gov/Request/dataRequestFAQ
NASA Cytokine Results	Crucian et al., 2014	LSDA (NASA)https://lsda.jsc.nasa.gov/Request/dataRequestFAQ

Experimental Models: Cell Lines		

Experimental Models: Organisms/Strains		

Oligonucleotides		

Recombinant DNA		

Software and Algorithms		

R package version 4.0.2		https://www.r-project.org/
Seurat 3.2.0	Stuart et al., 2019	https://github.com/satijalab/seurat
Patchwork 1.0.1		https://github.com/thomasp85/patchwork

Other		

BioAnalyzer	Agilent	D1000 HS
BD Rhapsody	BD Biosciences	633701
NovaSeq 6000	Illumina	n/a

## Highlights

- IL-6, IL-10, IL-1ra, CCL2, and CRP rose significantly on landing after a year in space
- IL-6 and IL-1ra are potential targets for pharmacological intervention in astronauts
- These cytokines are also associated with muscle regeneration in response to gravity
- Muscle-generated IL-6 drives classic signaling cascades in an anti-inflammatory role
