# Supplementary material for: Multi-omic, Single-Cell, and Biochemical Profiles of Astronauts Guide Pharmacological Strategies for Returning to Gravity
Source: Cell Rep. Author manuscript; Available in PMC 2022 Sep 5. (PMC9444344; doi:10.1016/j.celrep.2020.108429)
Supplement: 1 [file NIHMS1653534-supplement-1.pdf]

Cell Reports, Volume *33*

Supplemental Information

Multi-omic, Single-Cell, and Biochemical Profiles of Astronauts Guide Pharmacological Strategies for Returning to Gravity

Monica L. Gertz, Christopher R. Chin, Delia Tomoiaga, Matthew MacKay, Christina Chang, Daniel Butler, Ebrahim Afshinnekoo, Daniela Bezdan, Michael A. Schmidt, Christopher Mozsary, Ari Melnick, Francine Garrett-Bakelman, Brian Crucian, Stuart M.C. Lee, Sara R. Zwart, Scott M. Smith, Cem Meydan, and Christopher E. Mason

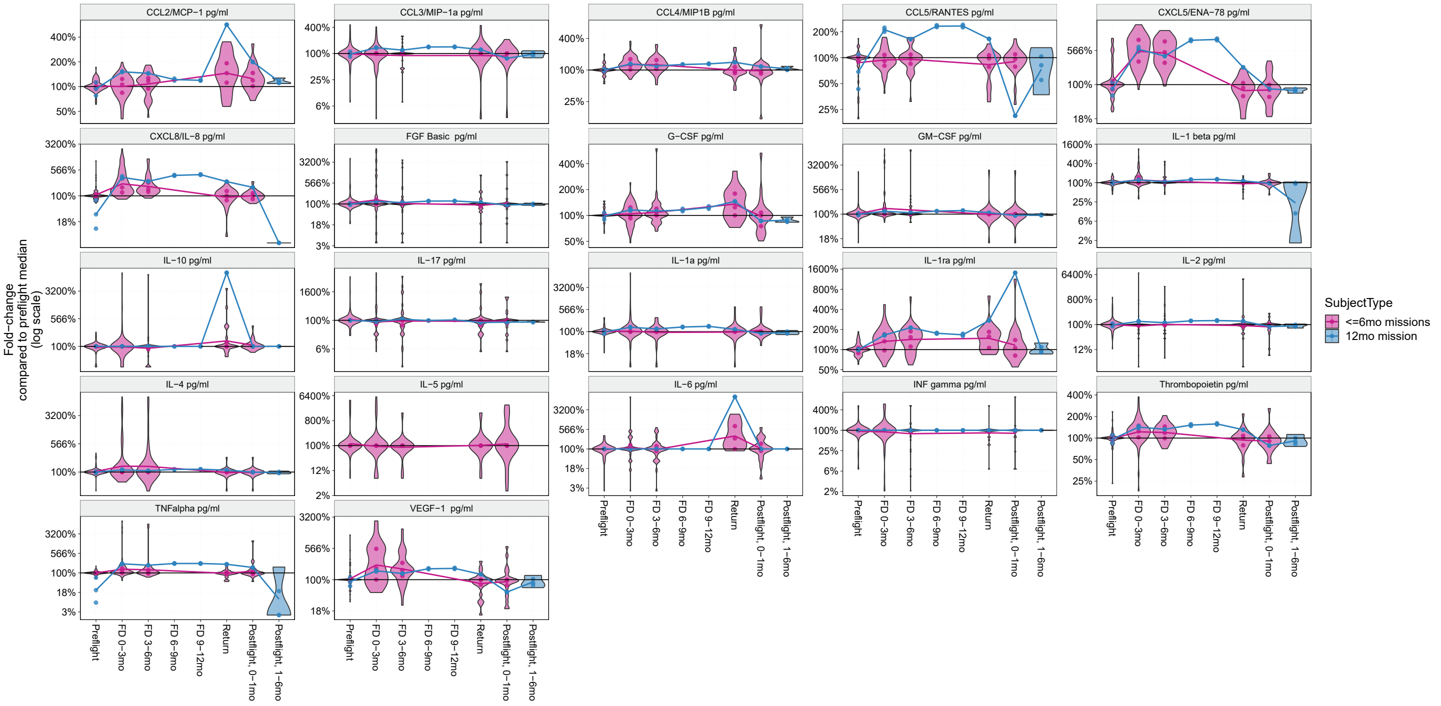

Figure S1. Comparison of biochemical profiles of the ISS year-long mission and 28 other astronauts from shorter prior expeditions, Related to Figures 1 and 3. All measurements were normalized per subject to their median preflight levels as baseline, including up to 180 days before launch. Return includes the day of landing and any measurements taken within 72 hours (Crucian et al., 2014).

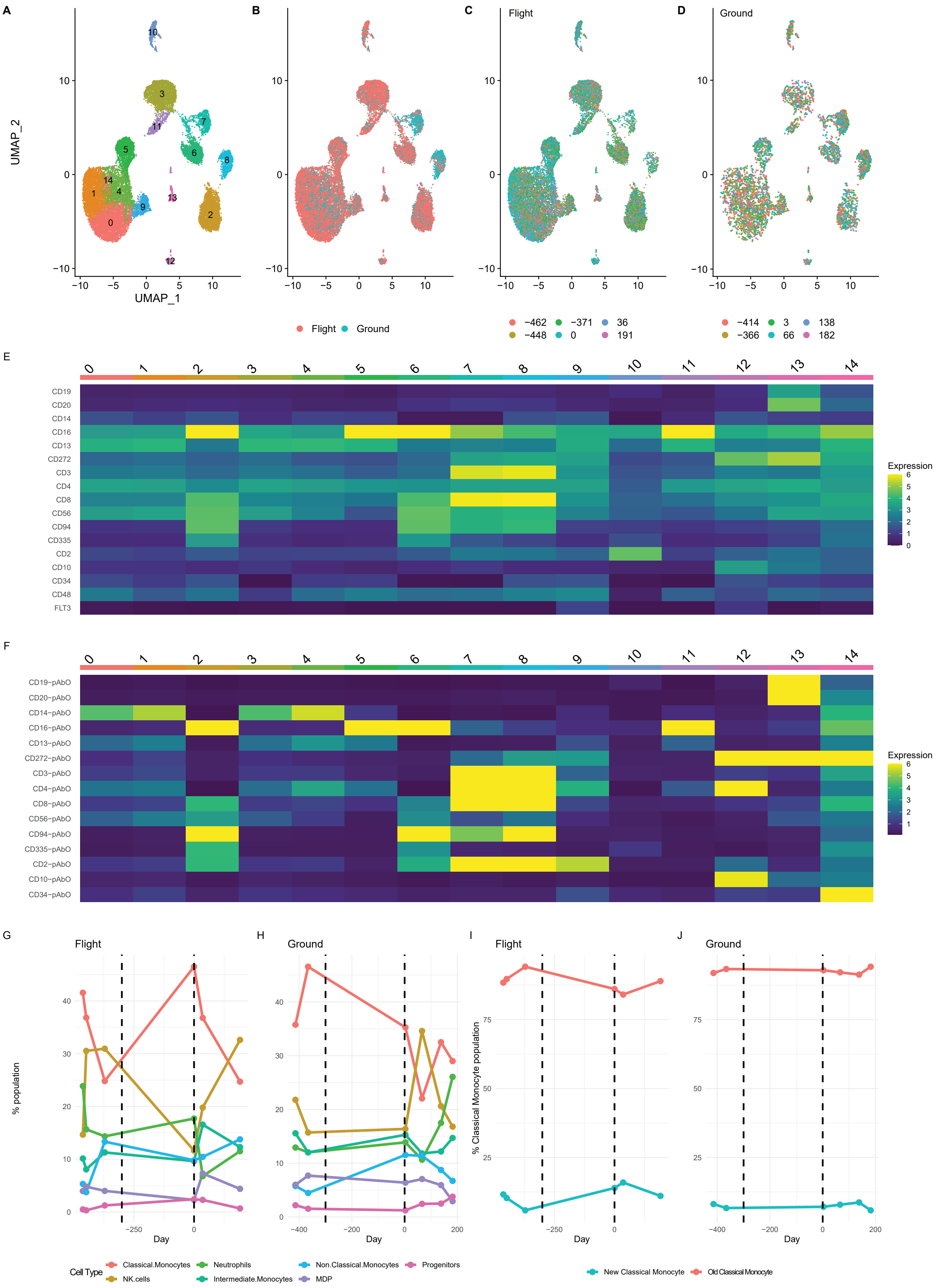

Figure S2. Identification of immune cell populations by single-cell clusters, Related to Figure 4. A-D. UMAP of cells showing Seurat clustering (A), flight cells vs ground cells (B), day collected for TW (C) and HR (D). E, F. Heatmap of mRNA (E) or epitope (F) expression of markers used to define clusters 0 through 14. G, H. Line graph showing population percentage as described in Figure 4B and 4C. I, J. Line graph showing population percentage as described in Figure 4N and 4O.

		Flight						Ground					
	Pathway	IL-6	IL-10	TNFR1	TNFR2	IL-4	GMCSF	IL-6	IL-10	TNFR1	TNFR2	IL-4	GMCSF
Pre-flight vs. Landing	Progenitors	1	1	1	1	1	0.000565	0.823643	0.121389	1	0.101981	1	1
	MDP	0.549434	0.304685	0.549434	0.549434	1	0.549434	1	1	1	1	1	1
	Classical Monocytes	9.33E-14	2.32E-14	0.696864	5.92E-13	8.74E-07	1	0.086876	1	0.002148	0.406016	1	0.406016
	Intermediate Monocytes	1	0.449427	0.449427	1	1	1	0.043771	1	0.344718	0.289127	0.034781	1
	Non Classical Monocytes	1	1	1	1	0.552069	1	1	1	1	1	1	1
	NK cells	0.000861	0.253094	0.023831	0.253094	8.76E-05	1	1	0.816669	0.162073	1	1	0.359889
	Neutrophils	5.55E-10	5.85E-05	0.153576	0.629836	0.629836	1	1	1	1	1	1	1
Landing vs. Post-flight	Progenitors	0.312378	1	1	1	1	1	1	0.634446	0.634446	0.634446	1	1
	MDP	1.16E-05	0.005182	0.004046	0.002452	0.143512	0.755399	1	1	1	1	1	1
	Classical Monocytes	1.24E-69	1	3.5E-114	7.11E-39	0.001433	2.49E-20	1	1	1	1	1	1
	Intermediate Monocytes	1.69E-17	0.250515	2.58E-23	0.001027	0.071489	0.049382	1	1	1	1	0.85377	1
	Non Classical Monocytes	8.55E-08	0.2965	1.87E-13	0.882445	0.282947	0.180002	1	1	1	1	1	1
	NK cells	1.85E-22	0.029426	1.32E-22	1	0.113603	0.563565	1	1	0.946365	0.398476	1	1
	Neutrophils	3.53E-12	1	2.35E-12	0.134565	0.158428	0.034656	1	1	1	1	1	1
Pre-flight vs. Post-flight	Progenitors	0.124018	1	1	1	1	0.067259	0.994656	1	0.626159	0.626159	1	1
	MDP	0.000647	0.287476	1.08E-05	0.044427	0.113525	1	1	1	1	1	1	1
	Classical.Monocytes	2.15E-39	4.83E-07	4.58E-98	5.92E-18	1	1.87E-17	0.35229	0.675991	0.35229	1	1	0.529807
	Intermediate.Monocytes	4.43E-17	0.004791	2.51E-29	5.71E-05	0.059215	0.013792	0.009891	1	0.026264	1	0.772013	1
	Non Classical Monocytes	5.53E-06	0.192995	3.18E-17	0.213208	1	0.192995	1	1	1	1	1	1
	NK cells	4.36E-16	0.431947	2.7E-19	1	0.68616	0.300154	1	0.357733	0.762752	0.277575	1	0.273976
	Neutrophils	0.000132	0.15973	1.14E-08	0.376385	0.376385	0.007775	0.337545	1	0.075488	1	1	1

Table S2. Wilcoxon rank sum p-values comparing pre-flight to landing, pre-flight to post-flight, and landing to post-flight for all of the pathways, Related to Figure 5. Days -462, -448 and -371 were classified as pre-flight, R0 was classified as landing, and R36 and R191 were classified as post-flight for these comparisons.
